# Assessing Substrate Utilization and Bioconversion Efficiency of Black Soldier Fly (*Hermetia illucens*) Larvae: Effect of Diet Composition on Growth and Development Temperature

**DOI:** 10.3390/ani14091340

**Published:** 2024-04-29

**Authors:** Simona Belperio, Arianna Cattaneo, Eleonora Nannoni, Luca Sardi, Giovanna Martelli, Sihem Dabbou, Marco Meneguz

**Affiliations:** 1 Department of Veterinary Medical Sciences (DIMEVET), University of Bologna, 40064 Bologna, Italy; simona.belperio2@unibo.it (S.B.); eleonora.nannoni2@unibo.it (E.N.); giovanna.martelli@unibo.it (G.M.); 2 Center Agriculture Food Environment (C3A), University of Trento, 38098 San Michele All‘Adige, TN, Italy; arianna.cattaneo@unitn.it (A.C.); sihem.dabbou@unitn.it (S.D.); 3 BEF Biosystems s.r.l., 10156 Turin, Italy; marco.meneguz@bef.bio

**Keywords:** black soldier fly, substrate, bioconversion, thermal-imaging camera

## Abstract

**Simple Summary:**

The increasing world population generates a huge volume of food waste. The use of black soldier fly larvae in the bioconversion of food waste is a new approach and an interesting example of a sustainable, circular economy approach. In this study, different food waste and residues (ranging from a vegetable to a carnivorous diet) were used as rearing substrates for black soldier fly larvae. The effects of different substrates on larvae development, bioconversion efficiency, and variations in surface temperatures were investigated. The results highlighted how an omnivorous diet provides performances comparable to the high-quality substrate diet (commercial chicken feed) and, therefore, can represent an effective strategy for rearing larvae while reducing food waste.

**Abstract:**

Black soldier fly larvae (BSFL) can utilize food by-products or residues for growth, benefiting farm animal’s diets’ production sustainability. The experiment aimed to assess the effect of different substrate compositions on larval growth, chemical composition, and substrate temperature. BSFL were allocated to one of the four diets (control, vegetable, carnivorous, and omnivorous) for the entire experiment (8 days). The temperature was measured twice daily using a thermal-imaging camera, and the accumulated degree hours (ADH) was calculated. The results showed that the larvae fed the vegetable diet exhibited a significantly reduced growth performance, with a biomass reduction of 26.3% compared to the control diet; furthermore, vegetable-fed larvae showed a lower dry matter content (−30% compared to the average of other diets) due to lower fat content (−65% compared to average of other diets). The nutritional composition of larvae fed an omnivorous diet was similar to larvae fed a high-quality substrate diet (control diet-chicken feed), indicating that the omnivorous diet could be an ideal solution for rearing BSFL larvae; however, the current European legislation prohibits the use of animal meal. The study also revealed that substrate temperatures did not have a discernible influence on larval growth, further emphasizing the importance of diet in BSFL rearing strategies.

## 1. Introduction

The increasing world population generates a huge volume of food waste (FW). According to the UNEP Food Waste Index Report, approximately 931 million tons of FW was generated in 2019 [1]. The United Nations included Goal 12.3 (“ensure sustainable consumption and production patterns”) among the 17 Sustainable Development Goals of the 2030 Agenda and aims to halve FW. This aligns with the overarching environmental impacts associated with food production and consumption. Management varies from country to country [2], and there are different recycling technologies of organic waste, i.e., incineration, landfill anaerobic digestion, and composting [3,4,5]. Inadequate management and lack of proper handling of FW have serious adverse effects on the environment and human health [6,7]. Addressing FW and guaranteeing food security is crucial for creating sustainable, healthy food [8]. In the context of waste valorization, a promising strategy is the use of FW as a substrate for mass-insect rearing [9]. The use of insects in the bioconversion of FW is a new approach and an interesting example of a sustainable and circular economy. The use of black soldier fly larvae (BSFL, *Hermetia illucens* L., Diptera: Stratiomyidae) in FW treatment has emerged as a key innovation [10] due to the high larvae production rate, low cost, and short life cycle. The voracity and development cycle of BSFL can also be exploited for the disposal of organic wastes with high environmental impact, such as manure from intensive livestock farming, agri-food industry waste, and urban solid waste [11,12]. The rapid expansion of BSF farming is also due to the benefits associated with the utilization of waste. Most of the current research on BSF investigates the larval stage, as it is the most suitable stage for recycling organic material, including FW, and produce biomass that can be used as feed [11,12,13,14]. The EU forbids the use of animal products (except milk, eggs, and derivatives) and municipal trash, particularly the organic portions of municipal solid waste, for industrial insect farming systems [15,16]. Nonetheless, it is legal to utilize vegetable food and by-product residuals as an insect’s food source [16,17]. Furthermore, Regulation 2021/1372 [18] removes specific restrictions on the utilization of animal proteins in animal feed. By endorsing the utilization of insect meal, these measures foster a more sustainable food chain.

Larvae can generate nutrient-rich biomass consisting, on average, of 22–45% crude protein, 26–40% fat, and micronutrients [19,20,21,22]. The nutrient compositions depends on the substrates provided and the rearing conditions [23]. Since nutrients affect the physiology, behavior, and growth of larvae, studies on nutrient requirements and, specifically, protein and lipid requirements are being conducted [24,25]. The ability of BSFL to convert waste into high- value nutrient biomass offers innovative economic opportunities for municipal solid waste management in different sectors [21,23,26].

Although environmental conditions (temperature, humidity, and aeration) do not need to be as controlled in larvae rearing as in adult rearing (where they are critical in determining copulation egg-laying activity and biomass production [27,28,29]), microclimatic control is also required during the early stages of insects’ lives [30,31]. Maintaining appropriate conditions and, in particular, temperature is the most effective method to obtain a successful bioconversion from BSFL [32,33]. Thermal summation models have been studied for understanding larvae developmental dynamics. These models allow researchers to determine the accumulated degree hours or degree day (ADH or ADD) required by a species to complete each developmental stage or reach specific larval lengths or weights [32,34].

To our knowledge, numerous studies on insect rearing substrates have investigated a single matrix of plant origin. Furthermore, at the European level, research on substrates of animal origin is also limited due to current regulatory constraints (Reg. 893/2017) [35]. This research aimed to study, through the comparison with a high-quality diet, the effects of combinations of different by-products, including those of animal origin, to evaluate which formulation could be most effective in BSFL larvae rearing, as well as in view of any future regulatory changes. Given the method of obtaining the FW materials for the vegetable and carnivorous diet, it was not possible to formulate diets providing equal amounts of nutrients. Consequently, we evaluated the growth performance, substrate utilization, and temperatures produced during the bioconversion activity carried out by BSFLs.

## 2. Materials and Methods

### 2.1. Colony

The study was conducted in the laboratories of the BEF Biosystems company (Turin, Italy). BSF adults were bred in a steel-frame cage (100 × 63 × 110 cm) covered with a mosquito net. The light source was an LED panel, with a wavelength suitable for BSF, as described by Oonincx et al. [36], with a photoperiod of light/night (12:12). BSF were maintained in a climate-controlled room with a temperature of 27 ± 1 °C and a relative humidity of 65 ± 5%. The adult flies were provided with water during their entire life, and wooden sticks were placed in the cage as an oviposition substrate. The wooden sticks were checked every day and replaced every second day. Such practice ensured larvae of the same age, thus facilitating development during the experiment. The eggs were collected from the colony using the methods outlined by Dortmans et al. [37]. Using a paintbrush, the eggs originating from multiple females were placed in plastic cups from wooden sticks and then transferred to the plastic boxes in which the experiment took place (size: 60 × 40 × 12 cm). After hatching, the larvae were fed a mixture of chicken feed and water until the test started.

### 2.2. Diet Formulation

Four experimental diets were formulated, with varying ratios of components depending on the diet type (Table 1):

1. Control diet (D1), a high-quality substrate (commercial chicken feed), according to the scientific literature [11,38].
2. Vegetable diet (D2), with by-products such as carrots, potatoes, and brewer’s spent grain. These by-products were mixed in a weight ratio of 1:1:1. The vegetable raw materials were sourced locally; the ratio used (1:1:1) was aimed to ensure an adequate supply of protein and sugars.
3. Omnivorous diet (D3), obtained by mixing a 1:1 ratio of the vegetable diet and the carnivore diet.
4. Carnivorous diet (D4), obtained by mixing ground beef epiglottis and cod pulp in a 1:1 ratio. For practical reasons, once the most suitable animal by-products available locally were identified, these were purchased from a Barf food trader (Tortona, AL, Italy).

### 2.3. Experimental Design

A pool of two kilograms of BSFL was passed through a vibrating sieve (2 mm, VibroWest MR 24//5.5.5, Milano, Italy) to eliminate small larvae. Then, three hundred 6-day-old larvae were weighed individually, using an analytical scale (U.S. Solid, Cleveland, OH, USA), to determine the average larvae weight (37.1 ± 9.02 milligrams). Sixteen blocks of 2000 larvae each were prepared using the average larvae weight. The 16 blocks were randomly assigned to one of the four diets (four replicates for each diet) and then weighted again and transferred to 16 plastic containers (32 × 23.5 × 11.5 cm). Furthermore, a seventeenth block was sampled by the initial larvae pool to conduct chemical analyses. The containers were fitted with a mosquito net on top to prevent larval escape or possible contamination. The containers were chosen specifically to result in the same height of substrate across treatments (~8 cm). 

The rearing facility maintained larvae at the temperature of 27 ± 1 °C, with 65 ± 5% relative humidity and a photoperiod (12:12). The amount of substrate in each replicate was calculated as 100 mg larva/day, as described by Diener et al. [11], throughout the trial period. A total amount of 2000 g of substrate was allocated in each container to ensure a ten-day food supply. The 16 plastic containers were randomly arranged according to a 4 × 4 design and shifted one position, twice daily, so as to guarantee similar conditions for all containers during the 8 days of the trial. From the second day onward, two ventilators provided constant ventilation to ensure the removal of moisture in excess for the entire experiment duration. On the third day, the mosquito lids were removed to allow for the homogeneous drying of the substrates.

### 2.4. Larval Growth

At the end of the experimental, the total biomass (larvae) and the residual rearing substrate were weighed (Kern, Balingen, Germany) for each container and then recorded based on wet weight. The following parameters were then calculated: growth rate (GR) and substrate reduction (SR).

GR (mg d^−1^) = (larvae final body weight (mg) − larvae initial body weight (mg))/days of trial (d);

SR (%) = [(distributed substrate (g) − residual substrate (g))/ distributed substrate (g)] × 100.

A sample of 100 larvae for each replicate was individually cleaned and photographed (Nippon Avionics CO., LTD, Shimamura-Building, Konobe-cho, Tsuzuki-ku, Yokohama-shi, Japan) orthogonally with a metric scale (mm). The ImageJ software package (v 1.50v, Bethesda, MA, USA) was used to examine the photographs. The length of the larvae was recorded from the mouthparts to the lower part of the last abdominal segment. 

### 2.5. Chemical Analysis and Utilization of Substrate Nutrients 

Substrates and larvae were frozen at −20° C and subsequently were analyzed in the laboratories of Animal Production and Food Safety service (SPASA) of the Department of Veterinary Medical Sciences (DIMEVET) of the University of Bologna, Italy. Samples of larvae and substrates were freeze-dried (Olsa, Milano, Italy) to remove all moisture contained and were ground using a shredder (Broyeur mélangeur MB 950G KINEMATICA, Malters, Switzerland). All samples were analyzed to measure crude protein (CP) [39] using a Kiedahl nitrogen analyzer (Gerhardt Vapodest50, Gerhardt Gmbh, Königswinter, Germany). For the nitrogen-to-protein conversion, the more precise N-factor of 4.67 proposed by Janssen et al. [40] was used instead of the standard conversion factor (6.25). Starch was determined according to AOAC Method 996.11, and ether extract according to AOAC Method 920.390020 [41]. Neutral detergent fiber (NDF), acid detergent fiber (ADF), and acid detergent lignin (ADL) were analyzed according to the method of Van Soestet al. [42], and ash was determined after 3 h of combustion in a muffle furnace at 550 °C (VULCAN 3-550, Dentsply Neytech Burlington, NJ, USA). 

The formula used to determine the conversion of each component of the biomass was calculated based on the initial and final analytical composition of the substrates:

x = (Ay − By)/Ay × 100,

where A and B were the initial (A) and final (B) weight (g) of the y component of the diet in the substrate. Utilization was calculated for dry matter, protein, fats, ash, starch, and non-structural carbohydrates.

### 2.6. Substrate Temperature and Accumulated Degree Hours

The temperature of the substrate of each box was recorded using a thermal-imaging camera (Nippon Avionics CO., LTD, Shimamura-Building, Konobe-cho, Tsuzuki-ku, Yokohama-shi, Japan). All containers were monitored twice a day (morning and afternoon) on days 1, 2, 3, 5, 7, and 8. The thermal images were taken directly above the container and included the entire surface. The maximum and minimum temperatures obtained from each thermographic picture were identified.

The software used to read the temperature was InfReC Analyzer NS9500 Standard (v. 5.0 C, Yokohama-shi, Japan). 

Similarly to previous work by Harnden and Tomberlin [32], we calculated the thermal summation model to obtain the accumulated degree hours (ADH) for each diet used. The ADH was calculated using the minimum, maximum, and mean temperature of the substrate; the critical temperature of the larvae LDT (low development threshold = 12 °C; [43]); and the duration of the experiment:

ADH = [temperature of substrate (°C) − LDT (°C)] × time (h),

where the substrate temperature was the minimum, maximum, and mean recorded by the thermal-imaging camera.

### 2.7. Statistical Analysis

Statistical analysis was performed using Statistica (StatSoft Inc., Tulsa, OK, USA, release 12, 2013). The Shapiro–Wilk test was used to verify normal distribution of the dependent variables for each combination of groups within-subject and between-subject factors. Levene’s test was used to verify the homogeneity of variances for each combination of groups of within- and between-subject factors. All data, except substrate temperature, were subjected to one-way analysis of variance (ANOVA), with diet as a fixed factor. Substrate temperature was analyzed using ANOVA, with diet and day of experiment as fixed factors. Tukey’s test was applied for pairs comparisons. Statistical significance was set at *p* < 0.05.

## 3. Results

### 3.1. Larval Growth

The results regarding the impact of the rearing substrate on BSF larvae development are reported in Table 2. All variables significantly differed (*p* < 0.01). In particular, group D2 showed a significantly lower larval biomass, larval weight, larval growth rate, and larval meal yield compared to the control (D1). This lower growth is confirmed by a greater quantity of residual biomass (significantly higher than in diets D3-omnivore and D4-carnivore). Overall, the D1 (control) diet performed the best, while D2 (vegetable diet) performed the worst in terms of larval growth and larval biomass production.

### 3.2. Larvae Chemical Composition

As shown in Table 3, the proximate composition of the larvae varied between the different diets, and, in particular, the protein, ash, and fiber contents were highly variable. 

Larvae fed a vegetable diet had a significantly lower protein content than those fed a carnivorous diet (*p* < 0.05). The fat content of the larvae was statistically lower in D2 compared to the other groups (*p* < 0.01), and the ash content was similar only in groups D3 and D4 and significantly lower compared to D1 and D2. Crude fiber was statistically different (*p* < 0.01), with D4 having the lowest and D2 the highest fiber content, while BSFL fiber fractions (NDF, ADF, and ADL) did not show any significant difference among treatments. Non-free extractive carbohydrates were lowest in D1 and highest in D2, with intermediate values for D3 and D4.

### 3.3. Substrates Chemical Composition

The results of substrate utilization are summarized in Table 4. All variables showed statistical differences between the groups. DM, OM, and mineral (ash) utilization was significantly (*p* < 0.01) lower in D1 than in other groups. Protein utilization was also lower in D1, with a significant difference from D3 (*p* < 0.01) and D2 (*p* < 0.05). Fat and starch degradation was lower in D4 than in all other groups (*p* < 0.01 utilization), and the utilization of non-structural carbohydrates was higher in D2 than in D1 and D3 (*p* < 0.01) and also in D4 (*p* < 0.05).

### 3.4. Substrate Temperature

**Figure 1:**
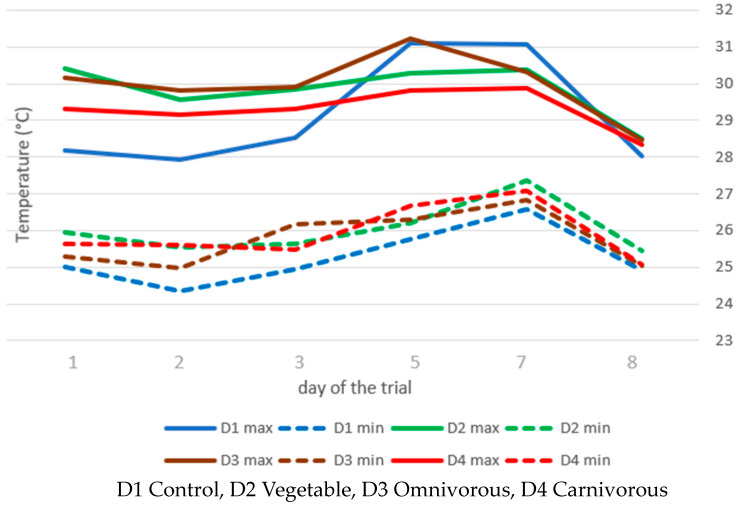
Daily average of maximum and minimum temperatures (°C) of rearing substrates during the trial (average of 4 replications per treatments).

The maximum and minimum temperatures recorded in the different substrates are shown in Figure 1. Overall, the D1 group exhibited a statistically significant difference, being lower compared to the other groups. A time effect was observed, with minimum temperatures being significantly higher on day 7 (*p* < 0.01) than on all other days (*p* < 0.01). Furthermore, the minimum and maximum temperatures recorded on day 8 were significantly lower (*p* < 0.01) compared to the two measurements on day 5 and day 7.

### 3.5. Accumulated Degree Hours

The maximum, minimum, and mean ADH values of BSFL reared on different diets are shown in Table 5. It was found that the diets had no significant effect on the minimum, maximum, and mean ADH values during the experiment period. The maximum ADH value was recorded for D3, while the minimum value was observed for D1. The mean ADH values ranged between 2964 and 3072. The significant lower temperature of the control diet (in particular, the minimum temperature) determined a trend (*p* < 0.1) for min ADH.

## 4. Discussion

One of the proposed strategies to reduce food waste and food loss is insect-based bioconversion [12,44]. By converting organic waste into useful nutrients, BSFL can play a significant role in recovering lost nutrients, as BSF is characterized by a low (i.e., very favorable) food conversion ratio. All diets used in this trial allowed larvae to grow and develop, although at different extents. BSFL reduced all four substrates and resulted in a different larval composition depending on the composition of the diet. If we exclude group D1 (the control diet), it is clear that D3 (omnivorous diet) administration resulted in better larval growth, larval biomass production, and substrate conversion. From a nutritional point of view, the content in dry matter, crude protein, and crude fat in D3 larvae is also similar to that in D1 larvae (Table 2 and Table 3). Our results are in agreement with those reported by Tschirner et al. [45], who found that the standard substrate group had the best results in terms of total larvae yield, individual larvae weight, and substrate consumption compared to the protein and fiber groups. Although the D2 diet showed similar (if not higher for organic matter and N-FE) substrate utilization coefficients than the control diet (Table 4), the substrate protein and fat contents were lower, resulting in less favorable larvae performances. This highlights that not only were protein and NFE limiting factors in this diet (D2), but so was fat. These findings are consistent with the trial conducted by Bellezza-Oddone et al. [24] where better growth performance was obtained from diets with a fat content of 4.5% (a value about three times higher compared to our study). The chemical composition of D2 larvae mirrors the chemical composition of the diet, in contrast to larvae fed the D3 and D4 diets, whose composition is similar to that of the larvae fed the control (high-quality) diet. Additionally, the D3 and D4 diets, despite being higher in protein and fat, did not lead to improved growth performances compared to the D1 diet, as also found by Nguyen et al. [46]. This result may suggest sugar content as a limiting factor in these diets. However, D4 larvae, due to both their higher DM to higher protein content compared to the other diets, allowed for the production of quantities similar to D1 in terms of larvae and protein yield. As suggested by Gold et al. [47], a well-balanced combination of substrates enhances growth and lowers variability, which is likely why larvae fed an omnivorous diet performed better in the present study. 

The substrate-utilization findings indicate that DM utilization, and particularly OM and CP, was lower in D1 compared to the other groups. Considering that D1 showed the best growth rates, it can be hypothesized that the substrate supplied may have been excessive in relation to the number of larvae present. Likely, this could be attributed to the higher initial content of mineral and micronutrients present in the complete chicken feed (D1), as demonstrated by the higher ash content. The utilization of starch was higher in D4 compared to the other groups, primarily because this diet had the lowest starch content. This result aligns with those of Barragan-Fonseca et al. [48], who observed that larvae yield was greater with diets with a high non-structural carbohydrate content. However, according to the growth performance of D1, it is clear that the larvae’s ability to grow is influenced by factors beyond the dietary amounts of proteins and carbohydrates. 

BSF is sensitive to several environmental factors, with the most important abiotic factor being temperature [48,49,50]. Numerous studies examined the effects of using organic side streams as feeding substrates or laboratory-reared meals at constant temperature on the life-history characteristics of BSFL [32,33,48,51]. As demonstrated also by Shumo et al. [52], both environmental temperature and substrate type significantly influence BSF larval development. The study by Ribeiro et al. [53] including various isolated vegetables and three different temperatures (20, 25, and 30 °C) showed that higher temperatures contributed to the rapid development of BSFL. In the present study, the control diet showed significantly lower minimum substrate temperatures compared to the other diets. On the other hand, larvae receiving the D1 diet exhibited the best growth performance. Therefore, the low temperatures did not appear to affect larval development in this study.

Another important aspect to consider is that, as the larvae grow larger, they produce more heat, but as the days go by, they feed less, and the heat decreases. Such a general shift in substrate temperature indicates changes in digestive activity and metabolism during larval ontogeny [54]. Our study agrees with the cited study, as it shows that substrate temperatures (minimum and maximum) increased with the age of the larvae, together with the increase in the amount of heat produced, but that the temperature began to decrease with increasing weight. As reported by Li et al. [55], the present study confirmed a similar trend in substrate temperature throughout the experiment. In the mentioned study, the maximum substrate temperature increased as the experiment progressed, peaked around the fourth day of the experiment, and then began to decrease. 

Harnden and Tomberlin [32], in an experiment where larvae were subjected to three different environmental temperatures (24.9 °C, 27.6 °C, and 32.2 °C), found that the minimum ADH (degree hours required for a species to complete each developmental stage or to achieve a specified larval length or weight) to complete larval development differed significantly for each diet and temperature. These authors conclude that the ADH for the completion of larval growth was lower for larvae reared on cereal meal and higher for larvae reared on pork. In our study, we applied a similar model to ADH and found that the type of diet had no effect on the temperature of the substrate during larvae development. This seems clear from the experimental design, which aimed to investigate whether the different diet composition of the diet has an effect on the temperature produced by secondary fermentation induced by larval activity. Different from Harnden and Tomberlin, in our trial, all replicates were kept at the same room temperature (27 °C).

The temperature recorded by the thermal camera reflects the temperature of the entire biomass surface. At the stage when the larvae prefer to remain on the surface in the dark rather than in the inner layers, this assessment could be even more interesting than measuring the temperature at a specific point of the substrate (for example, with a probe).

## 5. Conclusions

The utilization of black solder fly larvae in food waste bioconversion is an exciting example of a sustainable and circular approach. The different compositions of the tested diets did not determine any variation in the temperature (ADH) of the substrates. Larvae fed an omnivorous diet showed growth performances and a chemical composition comparable to those of animals receiving the control diet and better than larvae fed a diet based on plant by-products. This result is probably attributable to the better balance between sugars and proteins that characterizes the omnivorous diet. Although, according to European legislation, insects must be raised only on vegetal substrates, our results stimulate further studies on the nutritional properties of animal derivatives also in view of possible future regulatory changes.

## Figures and Tables

**Table 1:** Proximate composition of the four diets and young larvae (g kg^−1^ on a wet basis).

Variables		Initial Substrates			Young Larvae
	D1 Control	D2 Vegetable	D3 Omnivorous	D4 Carnivorous	
Humidity	722.0	695.9	744.2	754.8	697.4
Organic matter	239.2	284.6	238.5	230.2	-
Crude proteins	40.1	20.1	81.5	121.8	112.9
Crude Fat	14.9	9.4	34.4	54.3	31.8
Ash	38.8	19.5	17.3	15.0	50.6
Crude fiber	14.0	36.1	24.7	-	29.8
Neutral detergent fiber	48.1	68.8	85.1	-	43.8
Acid detergent fiber	22.9	59.0	35.5	-	37.6
Acid detergent lignin	7.60	18.7	12.5	-	7.2
N-free extractive ^1^	170.3	219.1	97.9	54.0	77.4
Starch	94.1	135.3	51.9	6.1	-

^1^ Calculated as 1000 − [ humidity + crude protein + fat + ash + fiber].

**Table 2:** Larval and residual biomass, larvae weight and length, growth rate, substrate reduction, dry matter larvae, and larvae meal yield of black soldier fly larvae reared on different substrates.

Parameter	D1 Control	D2 Vegetable	D3 Omnivorous	D4 Carnivorous	*p*-Value
Larval biomass (g) ^1^	376.9 ± 34.60 ^A^	278.0 ± 49.56 ^B^	335.1 ± 19.27 ^AB^	305.9 ± 24.21 ^AB^	*p* < 0.01
Dry matter larvae (%)	37.0 ± 7.13 ^A^	26.8 ± 5.27 ^B^	35.1 ± 5.50 ^AB^	44.4 ± 3.95 ^A^	*p* < 0.01
Larvae meal yield (g DM)	138.6 ± 21.92 ^B^	76.3 ± 29.35 ^A^	117.5 ± 18.29 ^AB^	135.4 ± 8.24 ^B^	*p* < 0.01
larvae weight (mg)	198 ± 21.4 ^A^	145 ± 10.2 ^B^	176 ± 35.5 ^AB^	161 ± 14.6 ^AB^	*p* < 0.01
Larvae length (cm)	1.7 ± 0.18 ^Aa^	1.4 ± 0.1 ^Bb^	1.5 ± 0.09 ^ABb^	1.4 ± 0.11 ^Bb^	*p* < 0.01
Growth rate (GR, mg d^−1^)	20.1 ± 4.2 ^A^	13.5 ± 2.5 ^B^	17.3 ± 4.5 ^AB^	15.5 ± 3.1 ^AB^	*p* < 0.01
Residual biomass (g)	380.1 ± 22.53 ^AB^	476.8 ± 53.48 ^B^	313.5 ± 14.92 ^A^	304.7 ± 26.27 ^A^	*p* < 0.01
Substrate reduction (%)	82.7 ± 1.02 ^B^	77.4 ± 2.85 ^A^	84.32 ± 0.75 ^B^	84.7 ± 1.31 ^B^	*p* < 0.01

^1^ g on a wet basis. ^A, B^ *p* < 0.01; ^a, b^ *p* < 0.05.

**Table 3:** Proximate composition (g kg^−1^ on a wet basis) of black soldier fly larvae reared on control (D1), vegetable (D2), omnivorous (D3), and carnivorous (D4) diets.

Black Soldier Fly Larvae					
Parameter	D1 Control	D2 Vegetable	D3 Omnivorous	D4 Carnivorous	*p*-Value
Crude protein	281 ± 4.9 ^ab^	273 ± 3.5 ^a^	288 ± 3.5 ^ab^	330 ± 6.8 ^b^	*p* < 0.05
Crude fat	261 ± 11.8 ^b^	97 ± 20.1 ^a^	303 ± 25.3 ^b^	256 ± 49.8 ^b^	*p* < 0.01
Ash	153 ± 2.8 ^c^	108 ± 13.8 ^b^	61 ± 7.9 ^a^	46 ± 2.1 ^a^	*p* < 0.01
Crude fiber	62 ± 3.3 ^ab^	91 ± 3.8 ^c^	65 ± 1.8 ^b^	58 ± 3.2 ^a^	*p* < 0.01
Neutral detergent fiber	128 ± 44.2	163 ± 34.3	127 ± 24.4	115 ± 12.5	*p* < 0.21
Acid detergent fiber	89 ± 6.7	151 ± 26.4	129 ± 53.3	139 ± 51.3	*p* < 0.18
Acid detergent lignin	11 ± 0.8	23 ± 2.7	25 ± 10.9	21 ± 10.4	*p* < 0.10
Non-free extractive ^1^	243 ± 37.0 ^a^	431 ± 47.9 ^c^	282 ± 34.9 ^ab^	310 ± 70.4 ^b^	*p* < 0.01

^1^ Calculated as 1000 − [ humidity + crude protein + fat + ash + fiber]. ^a, b, c^ *p* < 0.05.

**Table 4:** Utilization (%) of the substrate.

Parameter	D1 Control	D2 Vegetable	D3 Omnivorous	D4 Carnivorous	*p*-Value
DM ^2^	45.9 ± 4.52 ^A^	58.4 ± 4.29 ^B^	60.5 ± 2.02 ^B^	57.7 ± 2.40 ^B^	*p* < 0.01
OM	50.8 ± 4.31 ^A^	59.0 ± 4.18 ^B^	61.3 ± 2.27 ^B^	59.2 ± 2.64 ^B^	*p* < 0.01
CP	54.4 ± 5.78 ^Aa^	63.7 ± 5.76 ^ABb^	71.8 ± 3.71 ^Bb^	59.9 ± 2.32 ^ABab^	*p* < 0.01
CF	96.4 ± 1.89 ^B^	89.9 ± 3.38 ^B^	91.1 ± 3.24 ^B^	64.0 ± 5.70 ^A^	*p* < 0.01
Ash	15.8 ± 9.71 ^A^	49.2 ± 5.82 ^B^	49.3 ± 4.86 ^B^	34.7 ± 3.82 ^B^	*p* < 0.01
Starch	42.5 ± 4.36 ^B^	34.6 ± 12.62 ^B^	40.4 ± 5.45 ^B^	98.1 ± 0.37 ^A^	*p* < 0.01
NFE	49.6 ± 4.55 ^A^	61.4 ± 4.60 ^B^	43.6 ± 3.79 ^A^	52.7 ± 3.67 ^Ab^	*p* < 0.01

^2^ DM = dry matter; OM = organic matter; CP = crude protein; CF = crude fat; NFE = N-free extractive; ^A, B^ *p* < 0.01, ^a, b^ *p* < 0.05.

**Table 5:** Maximum, minimum, and mean accumulated degree hours (ADH, LDT = 12 °C) required by BSFL.

	D1 Control	D2 Vegetable	D3 Omnivorous	D4 Carnivorous	*p*- Value
ADH MAX	3360 ± 177.7	3430 ± 187.6	3456 ± 139.4	3315 ± 104.7	*p* = 0.27
ADH MIN	2568 ± 101.9	2713 ± 121.0	2647 ± 64.1	2690 ± 69.5	*p* = 0.10
ADH MEAN	2964 ± 136.3	3072 ± 149.8	3051 ± 98.1	3002 ± 82.9	*p* = 0.29

ADH MAX = ADH in relation to the maximum substrate temperature; ADH MIN = ADH in relation to the minimum substrate temperature; ADH MEAN = ADH in relation to the average substrate temperatures; ADH max, min, and mean are reported in Celsius degrees (°C).

## Data Availability

The raw data supporting the conclusions of this article will be made available by the authors upon request.
